# An Anatomist’s Anomaly Blank

**Published:** 1894-04

**Authors:** 


AN ANATOMIST'S ANOMALY BLANK, designed for the use of dissectors in recording any noteworthy anomaly that may be found, is a praiseworthy device of Dr. A. Hewson, of Philadelphia. P. Blakiston, Son & Co., Publishers, Booksellers, and Importers, Medical and Scientific Books, 1012 Walnut Street, Philadelphia.

The sheet has proper spaces for the student's name, the principal normal physical characteristics of the subject, and for anomalies of each of the principal organs or classes of tissues, in proper order and sequence. The proper recording of the anatomic peculiarities occurring in the thousands of dissections made in the country would eventually result in interesting and valuable scientific lessons.

